# Prevalence and Correlates of Risky Drinking Among the Oldest-Old in China: A National Community-Based Survey

**DOI:** 10.3389/fpsyt.2022.919888

**Published:** 2022-05-30

**Authors:** Yujia Qiu, Xiaozhen Lv, Tingfang Wu, Ying Zhang, Huali Wang, Bing Li, Xin Yu

**Affiliations:** ^1^NHC Key Laboratory of Mental Health (Peking University), National Clinical Research Center for Mental Disorders (Peking University Sixth Hospital), Peking University Institute of Mental Health (Sixth Hospital), Beijing, China; ^2^Beijing Anding Hospital, Capital Medical University, Beijing, China

**Keywords:** alcohol use problem, the oldest old, risky drinking, prevalence, national community-based survey

## Abstract

**Aims:**

To investigate the prevalence and correlates of risky drinking in Chinese elderly people aged 80 and over.

**Methods:**

Data were obtained from the Chinese Longitudinal Healthy Longevity Survey (CLHLS) conducted in 2018. A total of 10,141 respondents aged 80 years or older were included in this analysis. Risky drinking was defined as drinking > 2 drinks per day. The participants were divided into no risky drinking, past risky drinking, and current risky drinking groups. The prevalence of risky drinking, daily dosage, and type of alcohol beverages were assessed. The correlates of risky drinking were analyzed using logistic regression.

**Results:**

The prevalence of past and current risky drinking was 6.2 and 4.4%, respectively. A total of 12.2% of males and 2.1% of females reported past risky drinking, and 8.9% of males and 1.4% of females reported current risky drinking. The median of the daily dosage of the past risky drinking group was 4.5 and 4 drinks in males and females, respectively, and were 4 and 3.3, respectively, of the current risky drinking group. Strong liquor was the most popular alcohol beverage in all groups. Men who were older or had white-collar work were less likely to be past risky drinkers, while those with smoking in past or current or heart disease were more likely to be past risky drinkers. Women who smoked in the past were more likely to be past risky drinkers. Men with older age or living in the urban areas or with heart disease were less likely to be current risky drinkers. Women with higher education or with heart disease were less likely to be current risky drinkers. Women with current smoking were more likely to have current risky drinking.

**Conclusions:**

Our findings indicated that risky drinking among the oldest-old was not rare in China. The correlates of past and current risky drinking were different. Men and women had various correlates of risky drinking as well. Those with higher socioeconomic status seemed less likely to be risky drinking. More attention should be given to risky drinking among the oldest old, and sex-specific intervention may be needed.

## Introduction

China is facing great challenges in aging, as it has the largest and fastest aging population worldwide (1). In 2019, there were 164.5 million individuals aged 65 years old and over and 26 million aged 80 years old and over (the oldest-old) in China. It is estimated that there will be 115 million oldest-old individuals by 2050 (2). The rapid increase in the oldest-old population is of particular concern, since the oldest-old are the most susceptible to disease and disability (3), and they often need daily-living assistance as well as medical care, resulting in a heavy burden on the health care system, society, and families (4).

At the same time, rapid economic development and urbanization in China have also resulted in the increased alcohol use over the past 40 years (5). It is well-known that risky drinking affects the alcohol users' health, especially in those of old age. Previous studies reported that risky drinking was associated with increased morbidity, medical burden, and all-cause mortality (6). On the other hand, family members may hold the belief that risky drinking later in life does not exist or does not need treatment and will therefore overlook risky drinking among older adults (7). Health care workers may refrain from asking about risky drinking but focus more on their physical complaints (7). In fact, risky drinking seems to be not rare among the elderly. Germany reported 6.5% risky drinkers among older adults aged 75 years and over, including 12.1% male and 3.6% female (8). The Epidemiologic Catchment Area Study (ECA) in the United States estimates that alcohol abuse in the group aged 65 and over ranged from 1.9 to 4.6% for men and from 0.1 to 0.7% for women (9). The overall prevalence of heavy drinking among middle-aged and older adults was 7.23% in China (10). However, the evidence of the prevalence of risky drinking among the oldest-old is limited, and no evidence has been seen in the Chinese oldest-old population using a representative sample.

It is globally agreed upon that men are more likely than women to be risky drinkers due to cultural values, norms, and drinking patterns (11, 12). Chinese culture similarly has a long history of alcohol consumption among men. Therefore, it is necessary to explore the correlates of risky drinking in males and females. Except for sex and smoking, the correlates of risky drinking in previous studies were inconsistent. Some studies found that younger age, living with spouses, higher socioeconomic status, worse physical status, and greater anxiety were associated with risky drinking (5, 8, 13, 14), while other studies showed that lower education were associated alcohol use or depression was not associated with drinking (8, 15). Understanding the risky drinking of the older population can help medical practitioners to recognize and offer focused assessment.

The Chinese Longitudinal Healthy Longevity Survey (CLHLS) is a national community-based cohort study with the largest sample of oldest-old individuals in China. In this study, we investigated the prevalence of risky drinking and to explored its correlates based on the CLHLS 2018 survey.

## Methods

### Study Design and Participants

In this study, we obtained data from the CLHLS 2018 survey. The CLHLS is a national, ongoing cohort study of community-dwelling Chinese older adults from 1998. Follow-up occurred every 2–4 years, and the most recent (eighth) survey was completed in 2018. It is conducted in 866 highly diverse counties and cities selected from 23 of China's 31 provinces and covers ~85% of China's older population (4, 16). The CLHLS invited all centenarians in the sampled sites to voluntarily participate in the study and adopted a targeted random-sample design to interview approximately equal numbers of male and female non-agenarians, octogenarians, and young-old (aged 65–79 years) living near the centenarians to ensure representativeness (4). This design serves our aim of investigating the prevalence and correlates of risky drinking among the oldest-old in China. The surveys were administered through face-to-face interviews in participants' homes by trained interviewers with a structured questionnaire. There are details about CLHLS elsewhere (4, 16). The CLHLS study was approved by the Research Ethics Committee of Peking University (IRB00001052-13074), and all participants or their proxy respondents provided written informed consent.

Given that the present study focused on the current prevalence and correlates of risky drinking among the oldest-old, the data used were from the CLHLS 2018 survey. Participants who were aged 80 and over and completed the questions relating to alcohol drinking (including drinking status, drinking type, and dosage) were included. Participants who were younger than 80 and had no information about drinking were excluded.

### Measures

#### Definition of Risky Drinking

First, participants were grouped into never drinking, past drinking and current drinking groups according to their drinking status based on the question “Do you drink in the past?” and “Do you drink at present?”. Second, the past and current drinkers' daily dosage of pure alcohol consumption was calculated by multiplying the alcohol content (according to the alcohol content in different types of alcohol typically seen in China: strong liquor 53%, weak liquor 38%, beer 4%, grape wine 12%, rice wine 15%) and the amount drunk per day (the unit is “liang,” equivalent to 50 g). Third, the daily dosage was transferred into standard drinks, that is, per 10 g of pure alcohol equivalent to one standard drink (5). Risky drinking was defined as drinking alcohol above 2 drinks per day in old adults (17). Finally, participants with risky drinking were divided into past risky drinking and current risky drinking groups according to their drinking status, and others were grouped into the no risky drinking group.

#### Potential Correlates of Risky Drinking

Potential correlates of risky drinking in the oldest-old were selected according to previous studies (5, 13, 14, 18). The sociodemographic characteristics were sex, age, residence (urban, rural), ethnic group (han, others), marriage [in marriage, not in marriage (never married/widowed/divorced)], living arrangement (with families, alone), education (no schooling, schooling), occupation (no work, agriculture, white collar), and financial status (poor, middle, rich). Behaviors and health status were performing exercises (never, past, current), smoking (never, past, current), body mass index (BMI), falls (yes, no), hypertension (yes, no), diabetes (yes, no), dyslipidemia (yes, no), heart disease (yes, no), cerebrovascular disease (yes, no), gastrointestinal ulcer (yes, no), hepatitis (yes, no), Parkinson's disease (yes, no), epilepsy (yes, no), depressive symptoms (yes, no), anxious symptoms (yes, no) and sleep quality (good, not good). BMI was divided into four groups according to the Asian criteria: underweight-BMI < 18.5 kg/m^2^, normal weight-BMI > 18.5 to 23 kg/m^2^, overweight-BMI between 23–24.9 kg/m^2^, and obesity-BMI > 25 kg/m^2^ (19). Depressive symptoms were divided into two groups with a cutoff of 10 on the 10-item Center for Epidemiologic Studies Depression Scale (CES-D-10) (20). An anxious state was divided into two groups using the cutoff 10 of the Generalized Anxiety Disorder-7 (GAD-7) (21).

### Data Analysis

The descriptive results of sociodemographic variables, behaviors and health status, prevalence of risky drinking, daily dosage and type of alcohol were presented as frequencies (percentages) for categorical variables and means and standard deviations (SDs) or medians (p25, p75) for continuous variables according to the distribution of the variable. For group comparisons, one-way analysis of variance or non-parametric test was used for continuous variables, while the chi-square test was used for categorical variables. The factors with a *P*-value <0.1 in the univariate analysis and those identified in previous studies were included in the multinomial logistic regression analysis (Supplementary Table 1). Odds ratios (ORs) and 95% confidence intervals (CIs) were used in data interpretation for regression analyses. *P*-value < 0.05 was considered statistically significant. All analyses were performed with SPSS 20.0 (IBM SPSS Inc., Chicago, IL, USA).

## Results

### Description of Sociodemographic and Health Status

Of the 10,141 old adults, 4,077 (40.2%) males and 6,064 (59.8%) females, and the mean (SD) age was 92.32 (7.75). Overall, 55% lived in urban areas, 77.4% were not in marriage (never married/widowed/divorced), 76.8% were living with families, 63% had no schooling experience, and 53.3% had worked in agriculture. For the behavior and health status, 37% of the sample reported currently doing exercises, 12% were current smokers, 17% were obese, 25.3% had fall experience, 40.2% had hypertension, 7.7% had diabetes, 17.5% had heart disease, 11.3% had cerebrovascular disease, 49.4% had depressive symptoms, 11.6% had anxious symptoms, and 2.9% did not have good sleep. Compared to females, males were more likely to live in urban areas, be in marriage, live with others, be educated, be white-collar workers and have better financial status, do exercises currently, smoke, have a higher BMI, suffer cerebrovascular disease and Parkinson's disease but less likely to experience falls, suffer hypertension, depressive symptoms, anxious symptoms, and sleep trouble (see Table 1).

**Table 1:** Characteristics of 10,141 participants and compared between male and female.

		**Gender**		***p***
	**Total sample**	**Male**	**Female**	
**Variables**	**(*N* = 10,141)**	**(*N* = 4,077)**	**(*N* = 6,064)**	
**Sociodemographic characteristics**				
Age, year (Mean ± SD)	92.32 ± 7.75	90.61 ± 7.13	93.46 ± 7.94	**<0.001**
Residence, urban (%)	5,622 (55.0)	2,364 (58.0)	3,258 (53.7)	**<0.001**
Ethnic group, Han (%)	8,249 (81.3)	3,296 (80.8)	4,953 (81.7)	0.29
In marriage, no (%)	1,160 (77.4)	2,423 (60.0)	5,337 (88.9)	**<0.001**
Living with family member, yes (%)	7,784 (76.8)	3,187 (78.2)	4,597 (75.8)	**0.006**
No Schooling, yes (%)	5,461 (63.0)	1,249 (36.5)	4,212 (80.4)	**<0.001**
Occupation, *n* (%)				
No work	3,827 (37.7)	1,493 (36.6)	2,334 (38.5)	**<0.001**
Agriculture	5,410 (53.3)	1,920 (47.1)	3,490 (57.6)	
White-collar	904 (8.9)	664 (16.3)	240 (4.0)	
Financial status, *n* (%)				
Poor	1,140 (11.4)	426 (10.6)	714 (12.0)	
Middle	6,909 (69.1)	2,649 (65.8)	4,260 (71.3)	**<0.001**
Rich	1,949 (19.5)	952 (23.6)	997 (16.7)	
**Behavior and health status**				
Exercises, *n* (%)				
Never	5,516 (55.7)	2,090 (52.5)	3,426 (57.9)	
Past	716 (7.2)	168 (4.2)	548 (9.3)	**<0.001**
Current	3,665 (37)	1,723 (43.3)	1,942 (32.8)	
Smoking, *n* (%)				
Never	7,316 (73.1)	1,929 (47.8)	5,387 (90.3)	
Past	1,484 (14.8)	1,171 (29)	313 (5.2)	**<0.001**
Current	1,204 (12.0)	936 (23.2)	268 (4.5)	
Body Mass Index, *n* (%)				
Low	2,864 (28.5)	886 (21.9)	1,978 (32.9)	
Normal	4,160 (41.4)	1,767 (43.7)	2,393 (39.8)	
Overweight	1,318 (13.1)	650 (16.1)	668 (11.1)	
Obesity	1,713 (17.0)	740 (18.3)	973 (16.2)	**<0.001**
Fall, yes (%)	2,512 (25.3)	910 (22.8)	1,602 (27.0)	**<0.001**
Hypertension, yes (%)	3,739 (40.2)	1,459 (38.8)	2,280 (41.2)	**0.025**
Diabetes, yes (%)	687 (7.7)	292 (8.0)	395 (7.4)	0.291
Dyslipidemia, yes (%)	341 (3.9)	133 (3.7)	208 (4.0)	0.522
Heart disease, yes (%)	1,578 (17.5)	604 (16.5)	974 (18.1)	0.052
Cerebrovascular disease, yes (%)	1,015 (11.3)	486 (13.3)	529 (9.9)	**<0.001**
Gastrointestinal ulcer, yes (%)	398 (4.5)	147 (4.1)	251 (4.8)	0.119
Hepatitis, yes (%)	24 (0.3)	11 (0.3)	13 (0.2)	0.609
Parkinson, yes (%)	90 (1.0)	47 (1.3)	43 (0.8)	**0.025**
Epilepsy, yes (%)	26 (0.3)	11 (0.3)	15 (0.3)	0.857
Depressive symptom, yes (%)	4,458 (49.4)	1,787 (47.6)	2,671 (50.7)	**0.003**
Anxious symptom, yes (%)	1,035 (11.6)	326 (8.8)	709 (13.7)	**<0.001**
Sleep quality, good (%)	7,429 (97.1)	3,022 (97.7)	4,407 (96.8)	**0.023**

*SD, standard deviation. Data were presented as the mean ± SD, and n (%). P-values were obtained using the two-tailed two-sample t-tests for age, and using two-tailed Chi-square test for other variables*.

### Prevalence, Dosage, and Type of Risky Drinking

**Figure 1:**
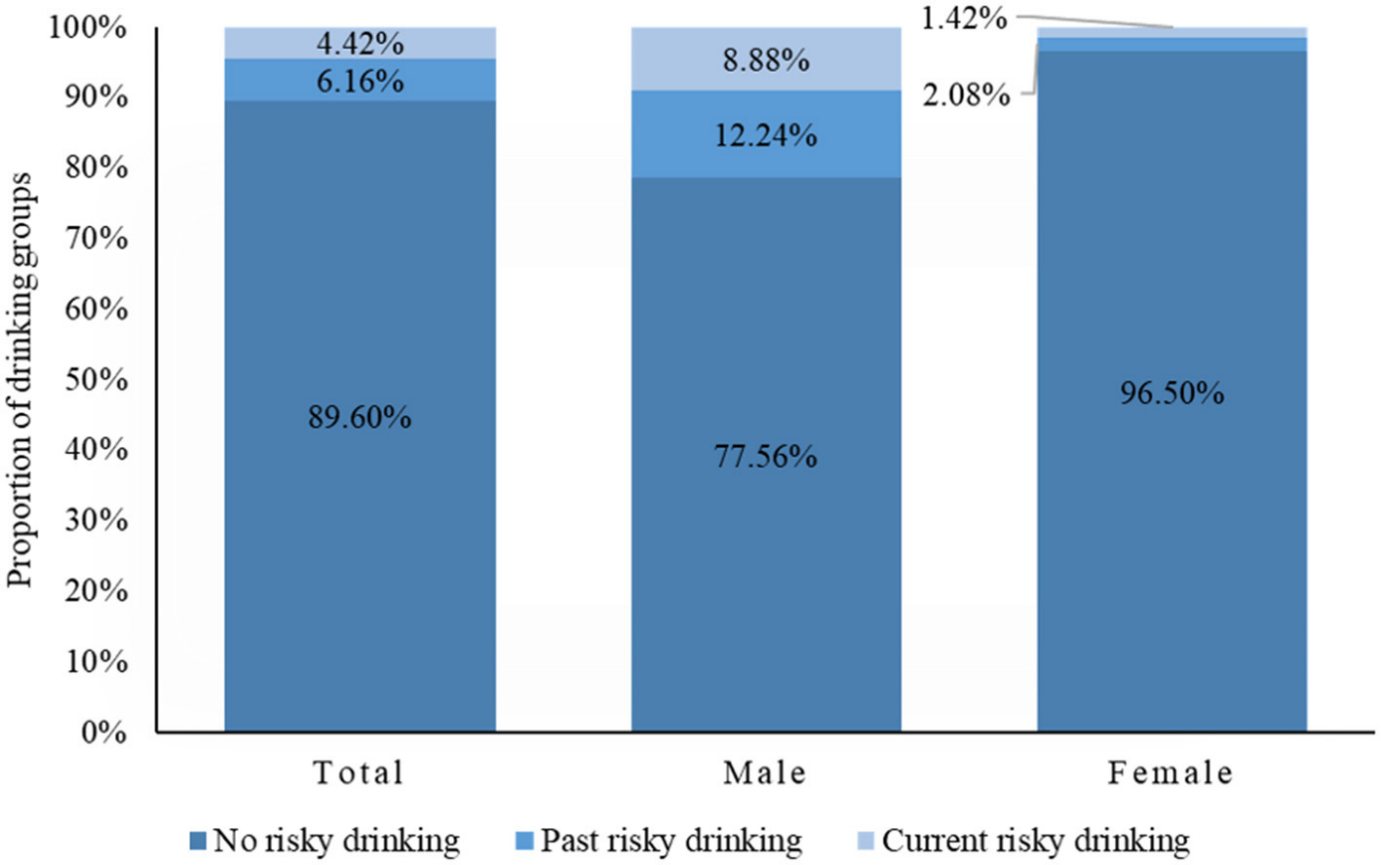
Proportion of different risky drinking groups among whole sample and different genders.

There were 77.2% of people who never drank, 11.2% had past drinking, and 11.6% were current drinkers. A total of 89.4% of participants reported no risky drinking, 6.2% reported past risky drinking, and 4.4% reported current risky drinking. Of the male respondents, 12.2% reported past risky drinking, and 8.9% reported current risky drinking. The corresponding figures for females were 2.1 and 1.4%, respectively. Males showed a higher prevalence of past and current risky drinking than females (*P* < 0.001) (Figure 1).

Among past risky drinkers, the daily dosage was 4.5 (4, 8) drinks for the total population, 4.5 (4, 8) drinks for males, and 4 (3, 8) drinks for females. Among current risky drinkers, the daily dosage was 4 (3, 6) drinks for total population, 4 (3.6, 6) drinks for the males, and 3.3 (3.4, 4.7) drinks for females. Among past risky drinkers, 481(77.0%) drank no <4 drinks per day, 390 (78.2%) for males and 91 (72.2%) for females. Among current risky drinkers, 310 (69.2%) drank no <4 drinks per day, 268 (74.0%) for males and 42 (48.8%) for females. The past risky drinking group drank more than the current group (Table 2).

**Table 2:** Daily dosage of alcohol drinking stratified by risky drinking group and sex.

	**Median drinks per day**	**Daily dosage group**		
		***n*** **(%)**		
	**(p25, p75)**	**drinks≤2**	**2 < drinks <4**	**≥4 drinks**
**Total sample**
Total	0	9,068 (89.5)	282 (2.8)	799 (7.8)
No risk drinking	1.5 (0.6,2)	9,068 (100)	–	–
Past risky drinking	4.5 (4, 8)	–	144 (23.0)	481 (77.0)
Current risky drinking	4 (3, 6)	–	138 (30.8)	310 (69.2)
**Male**
Total	0 (0,2)	3,216 (78.9)	203 (5.0)	658 (16.1)
No risk drinking	0	3,216 (100)	–	–
Past risky drinking	4.5 (4, 8)	–	109 (21.8)	390 (78.2)
Current risky drinking	4 (3.6,6)	–	94 (26.0)	268 (74.0)
**Female**
Total	0	5,852 (86.5)	79 (1.3)	133 (2.2)
No risk drinking	0	5,852 (100)	–	–
Past risky drinking	4 (3, 8)	–	35 (27.8)	91 (72.2)
Current risky drinking	3.3 (3,4.7)	–	44 (51.2)	42 (48.8)

*Data were presented as the median (p25, p75) and n (%)*.

Among those who drank in the past or at present, the most preferred alcohol beverage was strong liquor in all groups, which was 46.9, 46.4, and 46.6% in the no risky, past risky, and current risky drinking groups, respectively, as well as in males and females (Table 3).

**Table 3:** Type of alcohol beverages stratified by risky drinking group and sex among people who drank (*n* = 3,270).

					**Gender**							
	**Total sample**				**Male**				Female*			
	**Total**	**No risky drinking**	**Past risky drinking**	**Current risky drinking**	**Total**	**No risky drinking**	**Past risky drinking**	**Current risky drinking**	**Total**	**No risky drinking**	**Past risky drinking**	**Current risky drinking**
Strong Liquor	1,536 (46.9)	1,343 (46.9)	111 (46.4)	82 (46.6)	751 (48.8)	584 (49.3)	96 (48.0)	71 (46.4)	785 (45.2)	759 (45.3)	15 (38.5)	11 (47.8)
Weak Liquor	768 (23.4)	667 (23.3)	52 (21.8)	49 (27.8)	329 (21.4)	245 (20.7)	41 (20.5)	43 (28.1)	439 (25.3)	422 (25.2)	11 (28.2)	6 (26.1)
Grape wine	66 (2.0)	60 (2.1)	2 (0.8)	4 (2.3)	26 (1.7)	21 (1.8)	1 (0.5)	4 (2.6)	40 (2.3)	39 (2.3)	1 (2.6)	0 (0)
Rice wine	556 (17.0)	488 (17.1)	39 (16.3)	29 (16.5)	259 (16.8)	198 (16.7)	36 (18)	25 (16.3)	297 (17.1)	290 (17.3)	3 (7.7)	4 (17.4)
Beer	180 (5.5)	151 (5.3)	22 (9.2)	7 (4)	107 (7.0)	80 (6.8)	20 (10.0)	7 (4.6)	73 (4.2)	71 (4.2)	2 (5.1)	0 (0)
Others	153 (4.7)	152 (5.3)	13 (5.4)	5 (2.9)	57 (3.7)	57 (4.9)	6 (3.0)	3 (2.0)	96 (5.5)	95 (5.7)	7 (17.9)	2 (8.7)

*Data were presented as the percentage*.

### The Correlates of Risky Drinking

Compared with no risky drinkers, males (OR = 3.30, 95% CI: 2.33–4.66) and people who smoked (OR = 6.40, 95% CI: 4.68–8.76 for the past and OR = 3.26, 95%CI: 2.28–4.66 for the current) or had cerebrovascular disease (OR = 1.40, 95% CI: 1.01, 1.93) were more likely to have past risky drinking. In males, people with older age (OR = 0.97, 95% CI:0.95–0.99) and white-collar occupations (OR = 0.63, 95% CI:0.42–0.95) were less likely to have past risky drinking, but with heart disease (OR = 1.38, 95% CI:1.01–1.90) and smoking (OR = 6.33, 95% CI: 4.57–9.618 for the past and OR = 3.56, 95% CI: 2.34–5.40 for the current) were more likely to have past risky drinking. In females, those with past smoking were more likely to have past risky drinking (OR = 6.17, 95% CI: 3.50–10.86) (Table 4).

**Table 4:** Association between related factors and risky drinking in whole sample and different sex (*n* = 10,141).

**Variables**	**Past risky drinking**			**Current risky drinking**		
	**Total**	**Male**	**Female**	**Total**	**Male**	**Female**
	**OR (95% CI)**	**OR (95% CI)**	**OR (95% CI)**	**OR (95% CI)**	**OR (95% CI)**	**OR (95% CI)**
**Sociodemographic characteristics**					
Age	0.99 (0.97, 1.01)	**0.97 (0.95, 0.99)** ^*^	1.02 (0.99, 1.06)	**0.96 (0.94, 0.99)** ^*^	**0.95 (0.92, 0.97)** ^*^	1.01 (0.96, 1.07)
Male	**3.30 (2.33, 4.66)** ^**^	–	–	**5.82 (3.70, 9.14)** ^**^	–	–
Residence (Urban)	0.89 (0.68, 1.16)	0.84 (0.62, 1.14)	1.09 (0.64, 1.84)	**0.68 (0.50, 0.93)** ^*^	**0.67 (0.50, 0.90)** ^*^	0.88 (0.41, 1.88)
Marriage (Yes)	1.07 (0.79, 1.45)	1.05 (0.76, 1.47)	0.88 (0.32, 2.39)	1.38 (0.97, 1.97)	1.35 (0.93, 1.97)	1.53 (0.45, 5.18)
Schooling≥1	0.78 (0.61, 1.00)	0.81 (0.59, 1.11)	0.70 (0.31, 1.54)	**0.71 (0.53, 0.94)** ^*^	0.81 (0.57, 1.15)	**0.30 (0.09, 0.93)** ^*^
Occupation					
Agriculture	1.09 (0.79, 1.51)	0.96 (0.66, 1.40)	1.57 (0.80, 3.06)	0.83 (0.57, 1.20)	0.83 (0.55, 1.27)	0.82 (0.35, 1.94)
White collar	0.76 (0.47, 1.23)	**0.63 (0.42, 0.95)** ^*^	3.87 (0.96, 15.62)	0.76 (0.42, 1.37)	0.67 (0.37, 1.24)	2.81 (0.28, 27.84)
Financial status					
Middle	1.08 (0.70, 1.68)	1.29 (0.75, 2.20)	0.69 (0.32, 1.47)	1.29 (0.76, 2.18)	1.4 (0.77, 2.52)	1.08 (0.35, 3.31)
Rich	1.45 (0.88, 2.40)	1.63 (0.89, 2.99)	1.14 (0.47, 2.80)	1.14 (0.61, 2.14)	1.29 (0.6, 2.59)	0.64 (0.13, 3.12)
**Behavior and health status**
Smoking					
Past	**6.40 (4.68, 8.76)** ^**^	**6.33 (4.57, 9.61)** ^**^	**6.17 (3.50, 10.86)** ^**^	**2.63 (1.74, 3.97)** ^**^	**2.53 (1.64, 3.90)** ^**^	1.22 (0.15, 9.55)
Current	**3.26 (2.28, 4.66)** ^**^	**3.56 (2.34, 5.40)** ^**^	**2.08 (0.88, 4.92)** ^**^	**4.44 (3.44, 7.13)** ^**^	**4.34 (2.92, 6.44)** ^**^	**8.41 (4.42, 16.00)** ^**^
Body mass index					
Underweight	1.14 (0.83, 1.57)	1.00 (0.68, 1.48)	1.35 (0.77, 2.37)	1.11 (0.76, 1.62)	0.91 (0.59, 1.42)	2.27 (0.97, 5.31)
Overweight	1.06 (0.74, 1.53)	1.09 (0.72, 1.64)	0.94 (0.40, 2.22)	0.96 (0.61, 1.51)	1.01 (0.63, 1.61)	0.42 (0.05, 3.44)
Obesity	0.96 (0.67, 1.38)	1.09 (0.72, 1.64)	0.64 (0.27, 1.51)	1.36 (0.90, 2.06)	1.37 (0.88, 2.15)	1.62 (0.55, 4.75)
Hypertension (Yes)	0.98 (0.73, 1.31)	0.93 (0.66, 1.30)	1.12 (0.63, 1.98)	0.95 (0.67, 1.35)	0.93 (0.63, 1.36)	0.99 (0.41, 2.34)
Diabetes (Yes)	0.82 (0.54, 1.24)	0.87 (0.55, 1.37)	0.54 (0.16, 1.83)	0.57 (0.31, 1.07)	0.59 (0.31, 1.15)	0.45 (0.06, 3.46)
Heart disease (Yes)	1.28 (0.90, 1.82)	**1.38 (1.01, 1.90)** ^*^	0.86 (0.40, 1.84)	**0.49 (0.31, 0.78)** ^*^	**0.58 (0.35, 0.94)** ^*^	**0.23 (0.05, 0.97)** ^*^
Cerebrovascular disease (Yes)	**1.40 (1.01, 1.93)** ^*^	1.38 (0.96, 1.98)	1.48 (0.71, 3.09)	0.97 (0.67, 1.50)	0.98 (0.29, 3.28)	1.80 (0.49, 6.60)
Depressive state (Yes)	0.89 (0.68, 1.15)	0.90 (0.66, 1.21)	0.85 (0.50, 1.43)	0.96 (0.71, 1.30)	0.99 (0.71, 1.39)	0.80 (0.37, 1.73)
Anxious state (Yes)	1.19 (0.80, 1.77)	1.22 (0.75, 1.98)	1.16 (0.56, 2.39)	0.82 (0.47, 1.43)	0.64 (0.32, 1.29)	1.53 (0.55, 4.26)

OR, odds ratio; CI, confidence interval. *p < 0.05; **p < 0.001.

Compared with no risky drinkers, people who were older (OR = 0.96, 95% CI: 0.94–0.99), living in urban areas (OR = 0.68, 95% CI: 0.50–0.93), having schooling experience (OR = 0.71, 95%CI: 0.537–0.946) and having heart disease (OR = 0.49, 95% CI: 0.31–0.78) were less likely to have current risky drinking. Men (OR = 5.822, 95% CI: 3.705–9.148) and people who smoked (OR = 2.63, 95% CI: 1.74–3.97 for the past and OR = 4.44, 95% CI: 3.44–7.13 for the current) were more likely to have current risky drinking. In males, those of older age (OR = 0.95, 95% CI: 0.92–0.97), living in urban areas (OR = 0.67, 95% CI: 0.50–0.90), and heart disease (OR = 0.58, 95% CI: 0.35–0.94) were less likely to have current risky drinking. Males who smoked (OR = 2.53, 95% CI: 1.64–3.90 for past and OR = 4.34, 95% CI: 2.92–6.44 for current) were more likely to have current risky drinking. In females, those with higher education (OR = 0.30, 95% CI: 0.09–0.93) and heart disease (OR = 0.23, 95% CI: 0.05–0.97) were less likely to have current risky drinking. Females with current smoking were more likely to have current risky drinking (OR = 8.41, 95% CI: 4.42–16.00) (Table 4).

## Discussion

In our study, there were 6.2% past risky drinkers and 4.4% current risky drinkers. A total of 12.2% of males and 2.1% of females were past risky drinkers and 8.9% of males and 1.4% of females were current risky drinkers. Compared with females, males were more likely to have both past and current risky drinking. Among past and current risky drinkers, males had higher daily alcohol dosage than females. Except for female current risky drinkers, most risky drinkers drank no <4 drinks per day. Males of older age and white-collar occupation were less likely to have past risky drinking, but were more likely to have past risky drinking if they smoked or had heart disease. Females were more likely to have past risky drinking if they smoked in the past. Males of older age, living in urban areas, and having heart disease were less likely to have current risky drinking, but were more likely to have current risky drinking if they smoked. Females with educational experience were less likely to have current risky drinking, but if they smoke in the current, they were more likely to be current risky drinkers.

In our study, 11.6 % of elders were current drinkers and the prevalence of current risky drinking was 4.4% for all participants, 8.9% for males, and 1.4% for females. In a Chinese adult study, 68.2% were current drinkers, and 15% were risky drinkers (22). The prevalence of current risky drinking found in our study was also lower than that in several Western countries (21% for total, 20–12.1% for men and 9–3.6% for women) (8, 23, 24). The lower prevalence in our study compared with other data may be partly due to our older sample (average age 92.32 years). It is known that risky drinking is a behavior related to early mortality (8), namely, risky drinkers are more likely to die at a younger age than those with no risky drinking. Hence, survival bias may partially explain the lower prevalence of risky drinking in our study. In addition, the criteria for risky drinking and drinking are inconsistent in diverse studies and cultures (5, 17, 25, 26). In view of the different criteria for risky drinking and the lack of consensus about the criteria for risky drinking in the Chinese oldest-old (27), we chose 2 drinks as the cutoff based on previous literature (17). Most studies assessed drinking without distinguishing current from past risky drinking (8, 9, 23, 28, 29), while we differentiated past risky drinking from current risky drinking to clarify the association of correlates with past risky drinking. It was found that 70% non-drinkers were ex-drinkers, which may have a contaminating effect of drinking on health (25, 26). Thus, it is essential to classify risky drinking into past and current risky drinking. Consistent with previous reports (27, 30, 31), males had a higher prevalence of drinking or risky drinking. They drank faster with larger amounts than women, which mainly resulted from the cultural value and norms (12). Overall, our results indicated that past and current risky drinking in the oldest-old Chinese was not rare, especially among men. Considering risky drinking related to many health problems, it is necessary for families and professionals to pay attention to the oldest-old's drinking status.

In our study, the median number of drinks consumed by the oldest-old was 4.5 and 4 in the past and current risky drinking groups, respectively. It was found that among risky drinkers, most elders drank at higher dosages (28), which was consistent with our findings. We found that strong liquor was the most widespread alcohol beverage, and strong liquor and weak liquor, as the top two alcohol beverages, accounted for more than 60% of all alcohol beverages, while beer accounted for no more than 10%. In one study from the United Kingdom, it was shown that among a large sample of older people (75 years and over), relatively few elders drank more than 4 drinks per day, and half of them drank wine, 30% beer, and only 12% liquor (32). The difference in drinking patterns between different countries may be related to different cultures. The WHO reported that liquor accounted for 57% of alcohol beverages and beer accounted for 34% among young drinkers (aged 15 above) (33). The difference in preference for alcohol types in younger and older populations in China may result from young people bringing a more Western style of drinking into traditional drinking patterns. Consequently, the higher daily dosage and type of strong liquor should push health care and policy-makers pay more attention to the oldest old population and their drinking patterns.

In this study, older men were less likely to be risky drinkers both in the past and in the current, which is in line with similar findings from previous community-based studies (14, 28). One reason was that people may stop drinking after the negative outcome of drinking, which may increase with age (25, 26). In addition, the total body water and fat of elders decreased, metabolic ability of alcohol in the liver worsened, and blood alcohol concentration easily increased after drinking, resulting in decreased tolerance to alcohol, so elders may lower their consumption with age (34). Additionally, there may be more alcohol use limitations to drug combination as people get older (34). No association was shown between age and risky drinking among women. The sex difference may result from the lower dosage and drinking method of females (12).

In our study, it is shown that males living in urban areas and having been white-collar workers and females with schooling experience were less likely to have risky drinking. This was in accordance with another study of CLHLS, which showed that males living in rural areas were more likely to be drinkers (12). Data from the WHO showed that a generally higher level of economic wealth was greatly associated with increased levels of alcohol consumption and lower abstention rates (33). Another study also observed that older people were more likely to drink more if they had better socioeconomic status (35). The discordance may be partially due to culture and society. In rural areas in China, homemade rice wine is popular because of its affordable price and traditional customs (36, 37). In addition, many people in rural areas prefer herbal wine to treat diseases or symptoms, as the health care resources are less abundant (12, 36). The association between occupation and risky drinking in females was opposite to males, which may be due to the relatively small sample in females with working backgrounds, thus, it was not possible to test the schooling/no schooling differences.

Smoking, whether in the past or at present, showed a strong association with risky drinking, which has been well-documented in other studies (8, 32, 33). It is well-known that smoking and drinking frequently coexist (8, 38). We found that males with heart disease were more likely to have past risky drinking, which is in accordance with other studies (39, 40). This finding may indirectly support the evidence that risky drinking in the past increases the risk of heart disease. On the other hand, both males and females with heart disease were less likely to have current risky drinking. This may be due to the “sick quitters” who must stop drinking when they have a physical disease (39, 40). Addressing the correlates for risky drinking in the oldest-old population is of utmost importance and the public should not ignore this problem. Focus should be paid to these people with such correlates in practice.

The strengths of this study are as follows: (1) To the best of our knowledge, this is the first study to examine the prevalence and correlates of risky drinking among the oldest-old in China, with the largest representative sample of community-dwelling people aged 80 and over. (2) We divided risky drinking into past and current risky drinking, which provided the opportunity to describe and explore their correlates, respectively, and our results supported the necessity of this classification. (3) We have analyzed abundant possible correlates of past and current risky drinking.

There were several limitations in this study. The findings of this study could not be generalized to all populations except for community-dwelling oldest-old individuals. Moreover, the information was collected by self-report, which may result in recall bias, especially for past drinking dosage. However, previous evidence supported the validity of this self-report method (41). Additionally, as this is a cross-sectional study, the causal relationship of correlates and risky drinking cannot be drawn based on the present findings. We had no information about the medical treatment of the oldest-old which may be another correlate to risky drinking.

## Conclusions

Risky drinking of elders aged 80 years and over was not rare, especially in males. The correlates of past and current risky drinking were different. Men and women had various correlates of risky drinking as well. Those with higher socioeconomic status were less likely to be risky drinking. This study has filled the gap of risky drinking among the oldest old in China. More attention should be given to the issue of risky drinking among the oldest old, and sex-specific intervention may be needed.

## Data Availability Statement

The datasets presented in this study can be found in online repositories. The names of the repository/repositories and accession number(s) can be found at: https://www.icpsr.umich.edu/icpsrweb/DSDR/studies/36179.

## Ethics Statement

The studies involving human participants were reviewed and approved by the Research Ethics Committee of Peking University (IRB00001052-13074). The patients/participants provided their written informed consent to participate in this study.

## Author Contributions

YQ and XL designed concept, analyzed data, interpreted data, and prepared manuscript. XY designed concept, interpreted outcome, and reviewed manuscript. TW, YZ, HW, and BL interpreted outcome and revised the manuscript. All authors have read and approved the manuscript and ensure that this is the case.

## Funding

This study was supported by Self-exploration Project of National Clinical Research Center for Mental Disorders, Peking University Sixth Hospital (No. NCRC2020M10). The funding bodies had no role in the design of this study, data collection, analysis, and interpretation of data, and writing of the manuscript.

## Conflict of Interest

The authors declare that the research was conducted in the absence of any commercial or financial relationships that could be construed as a potential conflict of interest.

## Publisher's Note

All claims expressed in this article are solely those of the authors and do not necessarily represent those of their affiliated organizations, or those of the publisher, the editors and the reviewers. Any product that may be evaluated in this article, or claim that may be made by its manufacturer, is not guaranteed or endorsed by the publisher.
