# Supplementary material for: Prevalence and Correlates of Risky Drinking Among the Oldest-Old in China: A National Community-Based Survey
Source: Front Psychiatry. 2022 May 30;13:919888. doi: 10.3389/fpsyt.2022.919888 (PMC9195865; doi:10.3389/fpsyt.2022.919888)
Supplement: Supplementary file 1 [file Table_1.DOCX]

Supplementary File

# Prevalence and correlates of risky drinking among the oldest-old in China: a national community-based survey

# Yujia Qiu, Xiaozhen Lv, Tingfang Wu, Ying Zhang, Bing Li, Huali Wang, Xin Yu

# Supplementary Table s1 Characteristics of participants stratified by risky drinking and sex

|  | Total sample | | | |  | Gender | | | | | | | | |
| --- | --- | --- | --- | --- | --- | --- | --- | --- | --- | --- | --- | --- | --- | --- |
|  |  |  |  |  |  | Male | | | |  | Female | | |  |
|  | No risky drinking | Past risky drinking | Current risky drinking | *p* |  | No risky drinking | Past risky drinking | Current risky drinking | *p* |  | No risky drinking | Past risky drinking | Current risky drinking | *p* |
| Age, Mean±SD | 92.57±7.74 | 90.58±7.45 | 89.66±7.43 | **<0.001** |  | 90.98±7.11 | 89.81±7.15 | 88.46±6.82 | **<0.001** |  | 93.44±7.94 | 93.65±7.88 | 94.71±7.79 | 0.327 |
| Residence, urban(%) | 5058(55.8) | 346(55.4) | 218(48.7) | **0.013** |  | 1918(59.6) | 276(55.3) | 170(47) | **<0.001** |  | 3140(53.7) | 70(55.6) | 48(55.8) | 0.847 |
| Race, Han (%) | 7388(81.5) | 515(82.4) | 346(77.2) | 0.062 |  | 2612(81.2) | 406(81.4) | 278(76.8) | 0.122 |  | 4776(81.6) | 109(86.5) | 68(79.1) | 0.305 |
| In marriage, no(%) | 7099(79.1) | 395(63.9) | 266(60.3) | **<0.001** |  | 1949(61.2) | 282(57.2) | 192(53.8) |  |  | 5150(88.9) | 113(90.4) | 74(88.1) | 0.847 |
| Living with family member, yes (%) | 6947(76.6) | 502(80.3) | 335(74.8) | 0.063 |  | 2513(78.1) | 405(81.2) | 269(74.3) | 0.056 |  | 4434(75.8) | 97(77) | 66(76.7) | 0.932 |
| No Schooling, yes (%) | 5020(64.6) | 252(47.4) | 189(52.1) | **<0.001** |  | 961(35.5) | 165(39) | 123(42.4) | **0.034** |  | 4059(80.2) | 87(79.8) | 66(90.4) | 0.094 |
| Occupation |  |  |  |  |  |  |  |  |  |  |  |  |  |  |
| No work | 3425(37.8) | 233(37.3) | 169(37.7) | 0.608 |  | 1176(36.6) | 185(37.1) | 132(36.5) | **<0.001** |  | 2249(38.4) | 48(38.1) | 37(43) | 0.88 |
| Agriculture | 4845(53.4) | 325(52) | 240(53.6) |  |  | 1475(45.9) | 252(50.5) | 193(53.3) |  |  | 3370(57.6) | 73(57.9) | 47(54.7) |  |
| White-collar | 798(8.8) | 67(10.7) | 39(8.7) |  |  | 565(17.6) | 62(12.4) | 37(10.2) |  |  | 233(4) | 5(4) | 2(2.3) |  |
| Financial status |  |  |  |  |  |  |  |  |  |  |  |  |  |  |
| Poor | 1036(11.4) | 55(8.8) | 49(10.9) | **0.004** |  | 348(10.8) | 42(8.4) | 36(9.9) | 0.199 |  | 688(11.8) | 13(10.3) | 13(15.1) | 0.148 |
| Middle | 6325(69.8) | 416(66.6) | 312(69.6) |  |  | 2117(65.8) | 332(66.5) | 250(69.1) |  |  | 4207(71.9) | 84(66.7) | 62(72.1) |  |
| Rich | 1708(18.8) | 154(24.6) | 87(19.4) |  |  | 751(23.4) | 125(25.1) | 76(21) |  |  | 957(16.4) | 29(23) | 11(12.8) |  |
| Behavior and health status | | | | | | | | | | | | | | |
| Exercises |  |  |  |  |  |  |  |  |  |  |  |  |  |  |
| Never | 4932(36.8) | 345(56.7) | 239(54.9) | 0.08 |  | 1625(51.7) | 274(56.1) | 191(54.3) | 0.104 |  | 3307(57.9) | 71(58.7) | 48(57.8) | 0.374 |
| Past | 662(7.5) | 32(5.3) | 22(5.1) |  |  | 127(4.0) | 27(5.5) | 14(4.0) |  |  | 535(9.4) | 5(4.1) | 8(9.6) |  |
| Current | 3259(36.8) | 232(38.1) | 174(40.0) |  |  | 1389(44.2) | 187(38.3) | 147(41.8) |  |  | 1870(32.7) | 45(37.2) | 27(32.5) |  |
| Smoking |  |  |  |  |  |  |  |  |  |  |  |  |  |  |
| Never | 6998(78.3) | 177(28.5) | 141(32) | **<0.001** |  | 1750(55) | 91(18.3) | 88(24.6) | **<0.001** |  | 5248(91.1) | 86(68.8) | 53(63.9) | **<0.001** |
| Past | 1060(11.9) | 316(50.9) | 108(24.5) |  |  | 781(24.5) | 290(58.5) | 100(27.9) |  |  | 279(4.8) | 26(20.8) | 8(9.6) |  |
| Current | 884(9.9) | 128(20.6) | 192(43.5) |  |  | 651(20.5) | 115(23.2) | 170(47.5) |  |  | 233(4) | 13(10.4) | 22(26.5) |  |
| Body Mass Index |  |  |  |  |  |  |  |  |  |  |  |  |  |  |
| Low | 2635(29.3) | 138(22.3) | 91(20.3) | **<0.001** |  | 730(22.9) | 96(19.5) | 60(16.6) | 0.069 |  | 1905(32.8) | 42(33.3) | 31(36) | 0.554 |
| Normal | 3685(41.0) | 273(44.1) | 202(45.1) |  |  | 1381(43.3) | 221(44.8) | 165(45.6) |  |  | 2304(39.7) | 52(41.3) | 37(43) |  |
| Overweight | 1160(12.9) | 93(15) | 65(14.5) |  |  | 514(16.1) | 76(15.4) | 60(16.6) |  |  | 646(11.1) | 17(13.5) | 5(5.8) |  |
| Obesity | 1508(16.8) | 115(18.6) | 90(20.1) |  |  | 563(17.7) | 100(20.3) | 77(21.3) |  |  | 945(16.3) | 15(11.9) | 13(15.1) |  |
| Fall, yes (%) | 2241(25.3) | 171(27.7) | 100(22.8) | 0.184 |  | 708(22.5) | 127(25.8) | 75(21.2) | 0.199 |  | 1533(26.8) | 44(35.2) | 25(29.4) | 0.099 |
| Hypertension, yes (%) | 3354(40.4) | 243(42.0) | 142(34.1) | **0.027** |  | 1148(38.8) | 192(41.5) | 119(35.1) | 0.188 |  | 2206(41.3) | 51(44) | 23(29.9) | 0.108 |
| Diabetes, yes (%) | 628(7.9) | 43(7.7) | 16(4.0) | **0.016** |  | 239(8.4) | 39(8.7) | 14(4.3) | 0.300 |  | 384(7.6) | 4(3.5) | 2(2.6) | 0.74 |
| Dyslipidemia, yes (%) | 316(4.0) | 18(3.3) | 7(1.8) | 0.059 |  | 111(4.0) | 15(3.4) | 7(2.2) | 0.255 |  | 205(4.1) | 3(2.7) | 0 | 0.179 |
| Heart disease, yes (%) | 1425(17.7) | 115(20.3) | 38(9.4) | **<0.001** |  | 476(16.6) | 97(21.5) | 31(9.4) | **<0.001** |  | 949(18.3) | 18(15.8) | 7(9.1) | 0.093 |
| Cerebrovascular disease, yes (%) | 891(11.1) | 94(16.4) | 30(7.5) | **<0.001** |  | 382(13.4) | 78(17.1) | 26(7.9) | **0.001** |  | 509(9.9) | 16(13.8) | 4(5.5) | 0.169 |
| Epilepsy, yes (%) | 21(0.3) | 4(0.7) | 1(0.3) | 0.156 |  | 6(0.2) | 4(0.9) | 1(0.3) | 0.116 |  | 15(0.3) | 0 | 0 | 0.589 |
| Gastrointestinal ulcer, yes (%) | 366(4.6) | 23(4.1) | 9(2.3) | 0.078 |  | 121(4.3) | 18(4.0) | 9(2.4) | 0.287 |  | 245(4.8) | 5(4.4) | 1(1.4) | 0.409 |
| Hepatitis, yes (%) | 18(0.2) | 3(0.5) | 3(0.8) | 0.133 |  | 5(0.2) | 3(0.7) | 3(0.9) | 0.051 |  | 13(0.3) | 0 | 0 | 0.79 |
| Parkinson, yes (%) | 76(1.0) | 9(1.6) | 5(1.3) | 0.29 |  | 34(1.2) | 8(1.8) | 5(1.5) | 0.539 |  | 42(0.8) | 1(0.9) | 0 | 0.735 |
| Depressive symptom, yes (%) | 4018(50.2) | 263(45.5) | 177(41.1) | **<0.001** |  | 1436(48.8) | 207(44.6) | 144(41) | **0.009** |  | 2582(50.9) | 56(49.1) | 33(41.3) | 0.215 |
| Anxious symptom, yes (%) | 941(11.9) | 63(11.3) | 31(7.3) | **0.014** |  | 260(8.9) | 45(10) | 21(6) | 0.117 |  | 681(13.7) | 18(16.1) | 10(13) | 0.75 |
| Sleep quality, good (%) | 6669(97.2) | 448(97.0) | 312(96.6) | 0.799 |  | 2403(97.8) | 357(97.3) | 262(97) | 0.632 |  | 4266(96.8) | 91(95.8) | 50(94.3) | 0.503 |

Note: Data were presented as the mean±SD, and n(%). *P*-values were obtained using the one-way ANOVA for age, and using Chi-square test for other variables.
